# Study of Influence of Different Models of E-Learning Content Product Design on Students' Learning Motivation and Effectiveness

**DOI:** 10.3389/fpsyg.2021.753458

**Published:** 2021-09-20

**Authors:** Meng-Dar Shieh, Hsin-Yin Hsieh

**Affiliations:** Department of Industrial Design, National Cheng Kung University, Tainan, Taiwan

**Keywords:** product design, e-learning material, motivational belief, learning gains, learning effectiveness

## Abstract

As teachers provide one-way teaching demonstration or reference materials in class, students lack of the opportunities for direct operation. The provision of interactive e-materials could reduce the need for instructors to prepare complicated teaching aids and could deal with issues of climate and location. Learners could learn any time, anywhere, and learning could be reinforced by repeating the instruction with no need to disrupt timetables. The participants in the experimental teaching study were 275 product design students who engaged in e-learning for 15 weeks (3 h per week for a total of 45 h). The research results are summarized as follows: (1) Interactive teaching materials can enhance students' active learning styles so that, in the product design area, this method could reduce learning differences in students. (2) E-materials deliver knowledge using simple and specific images, animation, audio, and video, making learning interesting and relaxed for the product design students. E-learning is easy and practical and reduces learners' cognitive load. (3) Students' cognitive development and prior knowledge should be considered in the development of e-materials. Simple images, animations, text, and language could improve the attention and learning motivation of product design students and allow students to learn based on prior knowledge. A suggestion, based on the findings, is the application of various communication teaching models using e-materials to course work for the product design students, enabling discussion, analysis, concept formation, and problem-solving.

## Introduction

E-learning presents the functions of global communication, message switching, cooperative learning, and instant messaging. When applied properly, it deals with traditional media deficiencies and enhances the high application value of learning effectiveness. Furthermore, using the convenience of the Internet for “Internet-assisted teaching,” and placing the e-learning content prepared by teachers on websites, teachers could organize the content and Internet resources for learning Internet learners and for teachers, classmates. Learning management platforms and digital content are required for Internet-assisted teaching. The learning management platform is the online instruction tool for teacher–student interaction. Students can engage in discussion, observation, and cooperative group learning on the learning management platform. Teachers can provide teaching and guidance activities on the platform.

Digital content has various forms and characteristics. Simulation software can compress time, slow down the process, allow student participation, present safe experiments, and save money and other resources, making the impossible possible. E-learning material can be repeated in various forms and situations. Therefore, the form of digital content is a factor in online learning. Proper training is important to enable instructors to cope with different styles of learners. For maximum benefit, the content should match the learning styles, expectations, and approaches to instruction of the learners. Belonovskaya et al. (2020) mentioned that it is worth noting that continuing education concerns not only modern youth, but also older generations, who will also be affected by new technologies in the very near future. This study explores a new model of e-learning content aimed at product design students. Learning motivation and learning effectiveness are also investigated. Various communication teaching models for digital content are intended to help the students to deal with learning problems through discussion, analysis, and induction. The models could help students to establish correct concepts and to develop problem-solving abilities.

## Literature Review and Hypotheses

Teng (2019) mentioned that rich, lively, and changeable digital content was essential for making online learning attractive to learners. Online learning should be a combination of various forms of digital content. It should be adaptable to learners' characteristics and to the teaching goals. It was expected that learners could achieve the Internet-based teaching goals of activeness, interaction, simulation, and accumulation of learning. Demirkan (2019) pointed out the interaction between content and learners as a major characteristic of Internet courses, rather than the teaching content. Interaction with digital content on the Internet affects students' learning motivation. Scherer et al. (2019) found that interactive simulation content could strengthen learning motivation, reinforce the understanding of the learning process, and transfer to other similar situations, thereby avoiding classroom-based learning in challenging situations. In the simulation process, the delivery of content could be slowed down, and learners could construct personal learning concepts and develop validation and exploration mechanisms. The dynamic and interactive visual presentation allowed learners to correlate new material with previous experiences to develop an effective approach to improve conceptual learning. Therefore, the following hypothesis is proposed.

H1: The model of product design e-learning content would affect learning motivation.

Frolova et al. (2019) indicated that highly interactive computer-assisted instruction allowed computers to provide fast responses and gave users different degrees of learning control. The interactive learning control could be more efficient than computer-assisted instruction. Beatty et al. (2018) mentioned that teaching on the Internet could be preceded by a “classroom scene” and that there had to be “immediate spread” and “interactivity” to achieve effective teaching. Ugur (2019) studied constructive instructional design and considered that the learning system design had to match various instructional strategies. It had to consider the interaction between learners and learning content. For content design, multimedia, audio, and video could be utilized to induce students' curiosity and to achieve the objective of student–content interaction. Online learning could help students to learn using interactive digital content. Accordingly, the following hypothesis is proposed in this study.

H2: The model of product design e-learning content would affect learning effectiveness.

Yaman (2018) identified correlations between learning motivation and academic achievement. Learning motivation, as the motive force and guidance of learners' learning behavior and performance, could help learning behavior to advance continuously and to work toward specific directions or objectives. The apparent strength of learning motivation would directly affect individual learning and indirectly affect learning outcomes. Chartofili and Fokides (2019) found that students with high learning motivation presented definite goals and a strong desire to learn the content well. They had higher expectations of the outcome and better self-efficacy. It was also discovered that higher learning motivation would result in better effectiveness. Navarro-Pérez et al. (2019) found better performance in students with high learning motivation and better performance in students with intrinsic motivation than in those with extrinsic motivation. Consequently, the following hypotheses are proposed.

H3: Learning motivation presents significant and positive effects on learning effect in learning effectiveness.
H4: Learning motivation shows remarkable and positive effects on learning gains in learning effectiveness.

## Methodology

### Measurement of the Research Variables

#### Learning Motivation

Referring to Hwang et al. (2019), learning motivation is divided into (1) intrinsic orientation and (2) extrinsic orientation.

#### Learning Effectiveness

Referring to Chung et al. (2019), (1) learning effect and (2) learning gains are discussed.

### Research Objective and Sampling Data

In the experimental research, 275 college students from the Department of Product Design in Taiwan participated in a 15-week (3 h per week for a total of 45 h) using experimental teaching. On completion of the e-learning course, the students completed a questionnaire. The answers to this questionnaire were analyzed using SPSS, factor analysis, reliability analysis, regression analysis, and analysis of variance to test the various hypotheses.

### Analysis Method

Analysis of variance was used to identify the difference made by the model of product design e-learning content on learning motivation and learning effectiveness. The relations between learning motivation and learning effectiveness were further identified using regression analysis.

## Results

### Reliability and Validity Analysis

Learning motivation, with factor analysis, was extracted using the two factors of “intrinsic orientation” (eigenvalue = 2.164, α = 0.83) and “extrinsic orientation” (eigenvalue = 1.8626, α = 0.87). The cumulative covariance was 74.925%.

Learning effectiveness, with factor analysis, was extracted using the two factors of “learning effect” (eigenvalue = 3.673, α = 0.90) and “learning gains” (eigenvalue = 3.104, α = 0.93). The cumulative covariance was 84.066%.

### Effects of the Model of Product Design E-Learning Content on Learning Motivation and Learning Effectiveness

#### Difference Analysis of the Model of Product Design E-Learning Content in Learning Motivation

Analysis of variance was applied to identify the difference made by the model of product design e-learning content in learning motivation. The results show a significant difference in the product design e-learning content in intrinsic orientation. Interactive simulation (4.17) shows higher intrinsic orientation than streaming (3.69). Different models of product design e-learning content reveal remarkable differences in extrinsic orientation. Interactive simulation (4.34) appears higher for extrinsic orientation than for streaming (3.42). Therefore, H1 is supported.

#### Analysis of the Model of Product Design E-Learning Content in Learning Effectiveness

Analysis of variance was applied to identify the difference made by the model of product design e-learning content on learning effectiveness. The result shows a noticeable difference made by the model of product design e-learning content on learning effectiveness. Interactive simulation (4.26) indicates a higher learning effect than streaming (3.75), and interactive simulation (4.51) appears to bring about higher learning gains than streaming (3.91). Therefore, H2 is supported.

### Correlation Analysis of Learning Motivation and Learning Effectiveness

#### Correlation Analysis of Learning Motivation and Learning Effect

The analysis results in Table 1, testing H3, reveal the significant effects of intrinsic orientation (β = 2.362**) and extrinsic orientation (β = 2.147**) on learning effect. Therefore H3 is supported.

**Table 1:** Analysis of learning motivation on learning effectiveness.

**Dependent variable →**	**Learning effectiveness**			
**Independent variable↓**	**Learning effect**		**Learning gains**	
Learning motivation	β	*P*	β	*p*
Intrinsic orientation	2.362^**^	0.000	2.219^**^	0.000
Extrinsic orientation	2.147^**^	0.000	2.527^**^	0.000
*F*	26.417	35.304		
significance	0.000^***^	0.000^***^		
*R* ^2^	0.247	0.315		
adjusted *R*^2^	0.218	0.297		

** *p < 0.01 and*

*** *p < 0.001*.

*Data source: self-organized in this study*.

#### Correlation Analysis of Learning Motivation and Learning Gains

The analysis results in Table 1, testing H4, show the remarkable effects of intrinsic orientation (β = 2.219**) and extrinsic orientation (β = 2.527**) on learning gains. Therefore, H4 is supported.

## Discussion

Digital content delivers knowledge through images, animations, audio, and video and stresses simplicity and specificity. The aim is to make students feel that their learning is relaxed, interesting, and easy. Over-elaborate material could result in an excessive cognitive load. Effective learning should take the relationship among stimulus, response, and reinforcement into considerations. Under such an idea, learning content is divided into small units for learning in order and designed with the following principles. (1) Make definite learning goal and learner characteristics. (2) Use job analysis for dividing the teaching unit into several small units, and merely a small unit is preceded at a time. (3) Learning content is presented from simply to difficulty. (4) Feedback or reinforcement must be suitable for learners' cognition to give proper feedback or reinforcement. (5) Teaching materials should match learners' characteristics to offer massed practice or distributed practice. When approached correctly, interactive simulation content seems to have better learning effectiveness than teaching material involving complicated concepts and operations. When content design follows content concepts, and the level of difficulty in digital content takes the cognitive load into consideration, students' learning effectiveness is enhanced. Students' cognitive development and prior knowledge should be taken into account when digital content is being developed. When content is designed and using simple images, animations, text, and language to the greatest possible degree, based on their earlier experiences, students' attention and learning motivation will be enhanced.

## Conclusion

The research findings show that interactive simulation content could enhance students' learning effectiveness. In the experiment, the experimental group and the control group show notable differences in teaching content. In other words, interactive simulation content could enhance the learning effectiveness of teaching content. This could be the reference for future content design. Interactive simulation content provides simulated situations, allowing students to operate computer software. The human–computer interaction allows students not only to accept the information delivery through the sounds and images in the content but also to operate freely, to explore, and to observe. Students can access individualized instruction and be taught in accordance with their aptitudes. In this case, digital content with interactive simulation could reduce differences in learning styles. Such a method could be considered in the content design to reduce the differences among students with different learning aptitudes. Instruction with interactive simulation-based digital teaching materials could provide large amount of information for learners in a unit of time but might result in leaders' cognitive overload. To reduce leaners' cognitive load, unloading technique, decomposition technique, drill technique, cleanup technique, tagging technique, adjustment technique, redundancy elimination technique, synchronous technology, and personalized design could be applied in the interactive simulation-based digital teaching material design.

## Data Availability Statement

The original contributions presented in the study are included in the article/supplementary material, further inquiries can be directed to the corresponding author.

## Ethics Statement

The present study was conducted in accordance with the recommendations of the ethics committee of the National Cheng Kung University, Taiwan, with written informed consent being obtained from all the participants. All the participants were asked to read and approve the ethical consent form before participating in the present study. The participants were also asked to follow the guidelines in the form in the research. The research protocol was approved by the ethical committee of the National Cheng Kung University, Taiwan.

## Author Contributions

M-DS performed the initial analyses and wrote the manuscript. H-YH assisted in the data collection and data analysis. Both authors revised and approved the submitted version of the manuscript.

## Conflict of Interest

The authors declare that the research was conducted in the absence of any commercial or financial relationships that could be construed as a potential conflict of interest.

## Publisher's Note

All claims expressed in this article are solely those of the authors and do not necessarily represent those of their affiliated organizations, or those of the publisher, the editors and the reviewers. Any product that may be evaluated in this article, or claim that may be made by its manufacturer, is not guaranteed or endorsed by the publisher.
